# Photobiomodulation Therapy in the Management of Burning Mouth Syndrome: Morphological Variations in the Capillary Bed

**DOI:** 10.3390/dj8030099

**Published:** 2020-09-01

**Authors:** Giuseppe Alessandro Scardina, Sofia Casella, Giuseppa Bilello, Pietro Messina

**Affiliations:** Department of Surgical Oncological and Stomatological Disciplines, University of Palermo, 90121 Palermo, Italy; dott.casellasofia@gmail.com (S.C.); giuseppa.bilello@unipa.it (G.B.); pietro.messina01@unipa.it (P.M.)

**Keywords:** burning mouth syndrome, photobiomodulation therapy, videocapillaroscopy, diode laser

## Abstract

Burning mouth syndrome (BMS) is an idiopathic condition that manifests itself primarily with the onset of a burning sensation. The aim of this research was to perform photobiomodulation therapy (PBM) using a diode laser on the oral mucosa of BMS patients, followed by an objective evaluation of the morphological changes in the vascular bed underlying the mucosa using polarized light videocapillaroscopy. A group of 40 patients were included in the study. The patients were randomly divided into two groups (using simple randomization) as follows: 20 patients were assigned to the laser group and 20 patients were assigned to the placebo group. Each patient of the laser group received eight irradiations (with 4 Watt of power, wavelength 800 nm, energy 1200 Joules, irradiation time of 300 s, energy density 50 J/cm^2^, 60 mW continuous wave laser, and irradiance 180 mW/cm^2^), twice a week, blinded to the type of irradiation administered, for four consecutive weeks. The patients in the placebo group underwent the same sessions as the other patients, the only difference was the non-emission of the laser. An initial check of the vascular bed was performed with a polarized light videocapillaroscope. This was followed by treatment with a therapeutic diode laser and a subsequent check with a videocapillaroscope. We observed that in the group of patients who underwent laser therapy, there was a lasting improvement in symptoms. The capillary oral bed of patients in the placebo group did not show any statistically significant difference (*p* > 0.05). In the laser group we observed the following: in the buccal mucosa the diameter of the capillary had a reduction of 3 μm; in the upper lip mucosa, there was a reduction of 3 μm; in the lower lip mucosa, there was a reduction of 3 μm; and in the dorsal lingual surface, there was a reduction of 2 μm. An increase in capillary length was also obtained in all irradiated regions in the laser group patients (*p* < 0.05). PBM induces microcirculatory changes that are still present over a long period of time, such as an improvement in the clinical picture. The improvement in the symptoms has been correlated to the reduction of the capillary diameter. The placebo effect only led to a temporary improvement in symptoms that were unrelated to changes in the microcirculatory pattern.

## 1. Introduction 

Burning mouth syndrome (BMS) is an idiopathic condition that manifests itself primarily with the onset of a burning sensation in the oral mucosa [1,2,3]. Being part of a “syndromic” picture, the symptomatology can present heterogeneous and polymorphic patterns, i.e., the “burning” symptom can sometimes be accompanied by dysesthetic conditions of the oral mucosa, for example, alteration or loss of the sense of taste and dry mouth [2]. These symptoms inevitably alter the quality of life of the affected patient. 

From an epidemiological point of view, it is very difficult to assess affected patients, but it is known for certain that the pathology is widespread in the population (0.7–4.6%) and more frequent among females of postmenopausal age [3]. It is known that there is a close correlation between the manifestation of this burning sensation and the psychological conditions of the patient; the percentage of BMS cases that reflect psychosomatic mechanisms is high [1,3,4]. 

Some patients have a neurogenic phlogosis that could explain the burning sensation. In addition, it has been possible to observe alterations in the local microcirculation in these patients [5,6,7,8,9]. 

An instrumental analysis of the alterations that can be at the base of BMS, thus, causing the “burning” sensation, has been achieved using a videocapillaroscope [8,9]. From previous studies, it has been shown that by comparing the capillaries of healthy subjects with those of BMS subjects, it was possible to find a variation, above all, in one of the parametric data: the diameter of the capillary loop. In fact, the burning sensation can be explained by a “disturbance” in the local microcirculation [9]. 

Several studies in the literature have highlighted the efficacy of photobiomodulation (PBM) in BMS patients [10,11,12,13,14]. However, some studies have also noted a positive effect in non-laser groups or so-called placebo groups [10,15,16], although no study has been found in literature that evaluated the effects of PBM on microcirculation affected by irradiation. 

Our study aims to evaluate the effects of PBM in BMS patients. In particular, it seeks to evaluate if there is a correlation between PBM and the modification in the microcirculatory pattern, and if so, whether this modification of the vascular pattern can be correlated to the longer duration of the therapeutic effects in the PBM group as compared with the placebo group. We selected mainly post-menopause women because they are the most frequently encountered patients with BMS.

## 2. Methods

All subjects gave their informed consent for inclusion before they participated in the study. The study was conducted in accordance with the Declaration of Helsinki and the protocol on oral microcirculation evaluation in healthy subjects and patients affected by oral and systemic diseases was approved by the Ethics Committee of Policlinic Palermo N#02/2010.

### 2.1. Participants and Pain Evaluation

Forty females who were of post-menopausal age (mean age 62.06 ± 3.1) were enrolled in the pilot study. At the time of recruitment, they were given a form to complete regarding various points (personal data; habits of oral hygiene; lifestyle factors; level of pain on the VAS (visual analog scale), NRS (numeric rating scale), and pain quantization; and the areas in which videocapillaroscopic evaluations were recorded). The inclusion criterion was a diagnosis of BMS [17,18,19,20]. All the women had burning sensation and a total absence of other oral pathologies (candidiasis, lichen planus, glossitis, periodontitis, etc., i.e., patients who had healthy mucosa) or systemic pathologies (vitamin and mineral deficiencies, diabetes, autoimmune disorders, etc.). Moreover, smokers, patients who had reported a previous appearance of mycosis, hypertensive patients (because of collateral effects of their pharmacological therapy), and, in general, patients submitted to daily pharmacological treatments were excluded from the study [1,2,3,5,9,18]. The protocol for carrying out the procedure was standardized for all the patients. Patients were made aware of the aim of the study and of any, if transitory, adverse effects it could have through informed consent to treatment. Respect for ethical and bioethical principles was guaranteed (IRB approval). 

First, the patients were given an evaluation form which included the administration of the VAS and NRS scale to allow the patient, in a subjective way, to numerically quantify the burning sensation in the following 4 specific regions: the upper labial mucosa, the buccal mucosa, the dorsal lingual surface, and the lower labial mucosa. They were asked to quantify the burning sensation in these four regions specifically because it was easier to evaluate the blood vessels there. Given that the most important variations were expected to be found in the diameters and lengths of the capillaries, no periosteum regions were evaluated. 

### 2.2. Videocapillaroscopy Examination

Once the burning was quantified, the videocapillaroscopic examination (Horus) was performed in the same regions to evaluate the morphology of the capillary bed during the burning symptomatology, evaluating both parametric data (capillary loop length, capillary loop diameter, capillary density, and capillary tortuosity) and non-parametric data (the presence of capillaries with particular morphology) [9]. An intraoral instrumental examination was performed with the subject in a sitting position, with the same light source (a 6500 °K medical neon light), at the same room temperature 23 °C, in the morning, and by the same operator. It is important to emphasize that the parametric data originated from software related to videocapillaroscopy using a dedicated measuring instrument, with each optical magnification corresponding to an exact value of metric pixels in the scanned image. The capillaroscope used was the most recent generation videocapillaroscope. The apparatus consists of a handpiece, 3–4 cm in diameter and 11 cm long, weighing 180 g, equipped with microfocus to bring the area being examined into exact focus and allow the operator to work easily in all directions. At the working end, there is a high-resolution camera equipped with a contact scanning system and epiluminescence immersion; microillumination provided by a white light LED; high brightness achromatic optics with micro focus; and a 30× optical zoom with the possibility to get up to 150× magnification through the magnification module, with the horizontal and vertical resolution down to 0.4 μm (Videocap 100 VCS). The nonworking end was connected directly to a computer via a single USB cable (3.0), through which it received power and provided the digital information. The company provided the software (Videocap 100 VCS) that made acquiring and reporting data both fast and easy.

### 2.3. Laser Irradiation

**Figure 1:**
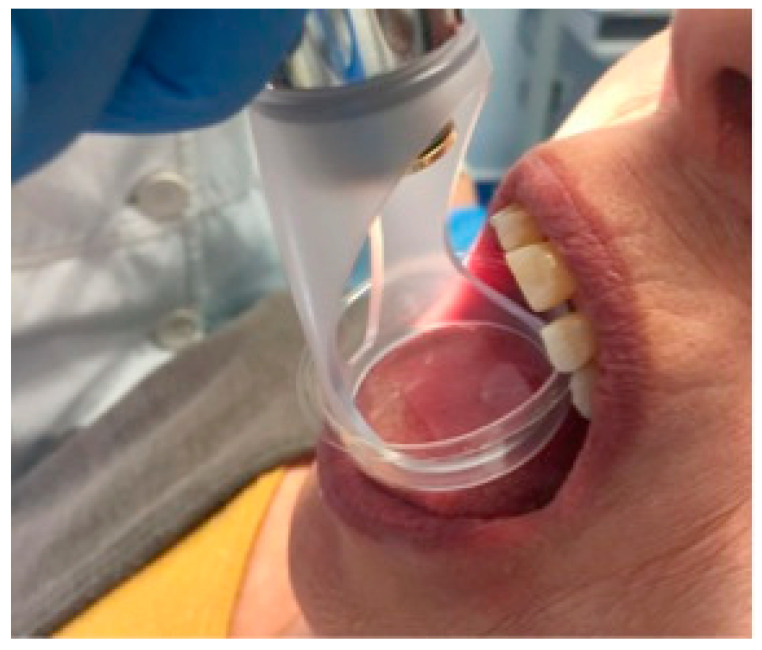
Application of photobiomodulation therapy on the dorsal lingual surface.

**Figure 2:**
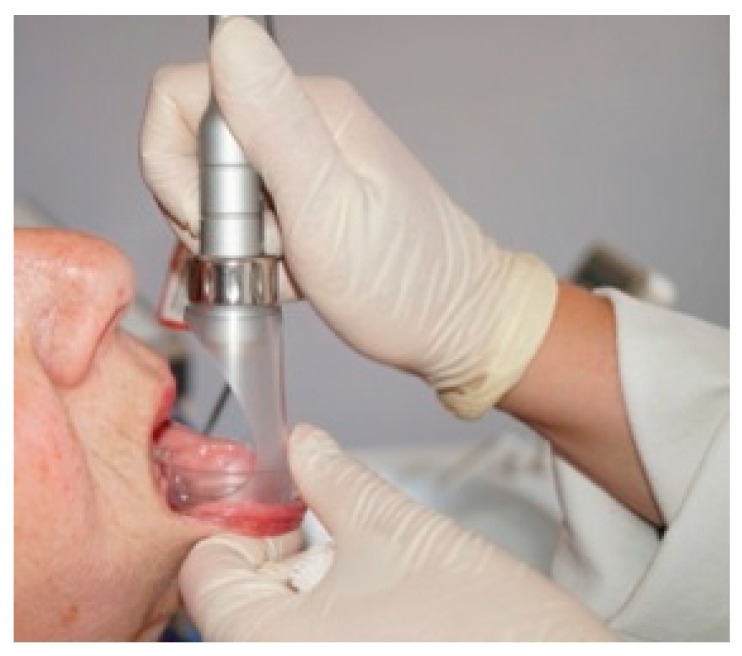
Application of photobiomodulation therapy on the lower lip surface.

The patients were randomly divided into two groups (using simple randomization). A group of 20 patients were assigned to the laser group and 20 patients were assigned to the placebo group. The study was conducted as a double-blind study, therefore, neither the evaluator (G.A.S.) nor the patients were aware of the subdivision into two groups. The patients in the placebo group underwent the same sessions as the other patients, the only difference was the non-emission of the laser, although the sounds and the switching on of the device were identical to the emission of the laser, therefore, the patients could not notice the non-emission of the laser. As for the capillaroscopic sessions, these were identical for the two groups, and the operator did not know if the patient was in the placebo group or not. Subsequently, a diode laser (BioLase Epic 10) was electively applied in the four above-mentioned areas (in these areas, the laser was applied using a “scanning” technique and not in the treated points). The diode laser was applied to each of the four areas (Figure 1 and Figure 2) with 4 Watt of power, wavelength 805 nm, energy 1200 Joules, irradiation time of 300 s, energy density 50 J/cm^2^, 60 mW continuous wave laser, and irradiance 166.7 mW/cm^2^ as per protocol. The application of the laser was performed with a handpiece, holding the handpiece exactly 4 cm from the mucosa, which was the distance necessary for the collimation of the light beam [18,19]. The distance was precisely maintained thanks to the spacer provided with the diode laser.

**Figure 3:**
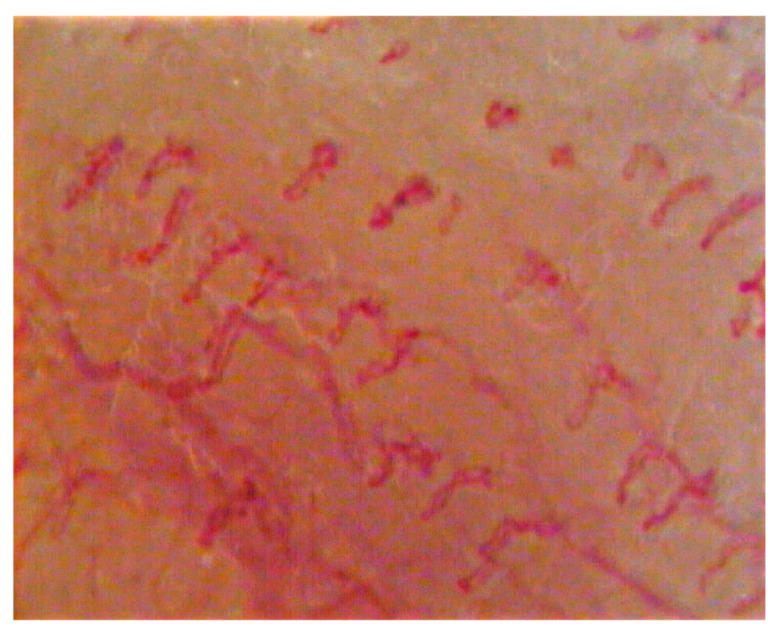
Capillaroscopic evaluation in burning mouth syndrome (BMS) patients before laser application.

**Figure 4:**
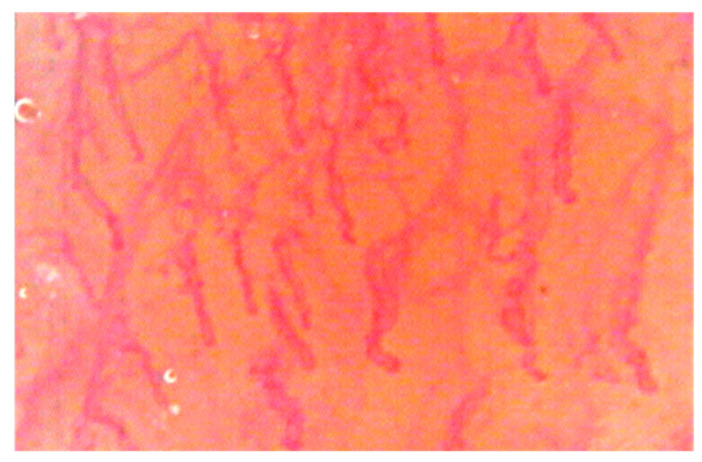
Capillaroscopic evaluation in BMS patients after laser application.

Once the diode laser was administered (both in the laser group and in the placebo group) the videocapillaroscopic evaluation was performed at the irradiated sites, in the same order (Figure 3 and Figure 4).

Experimental design: the treatment was performed with a diode laser on the upper labial mucosa (300 s), and then the treatment was performed on the buccal mucosa (300 s) and a videocapillaroscopic examination was performed in the two above-mentioned regions in order to evaluate the immediate effects of the diode laser, which appeared after 7–8 min, so as to intercept the most “probable” period in which variations of the capillary bed manifested themselves. Then, the other two regions (the dorsal lingual surface and the lower labial mucosa) were irradiated (300 s) and a videocapillaroscopic evaluation conducted immediately afterwards. 

Subsequently, the VRS and NRS scales were re-administered and the symptom recorded after treatment. All this protocol was repeated twice a week, for 4 weeks, for a total of 8 sessions (T#1/T#8). After a 60-day window, the patients in both groups were re-evaluated both with regard to the pain symptom, using the same scales (VAS and NRS), and with regard to the oral vascular pattern in the same oral sites that had previously been studied.

Data collection and statistical analysis were performed with the help of the Open Office 4.1.3 program. The mean (capillary length, capillary diameter, and capillary density) and prevalence (capillary tortuosity and capillary morphology) for the four irradiated and non-irradiated regions examined in the study were calculated for the 40 patients included in the study, in order to understand the mean values of these parameters in the population affected by BMS. Once the overall mean parameters were obtained, a further mean and a further prevalence were recalculated by relativizing them to the different time instances in which the treatment was performed, that is, in eight instances of time that represented the individual sessions. Once these parameters had been obtained at the various instances of time, a subdivision in quantiles (and more particularly in quartiles, identified as T#2 after treatment, T#4 after treatment, T#6 after treatment, and T#8 after treatment) was performed in which the variation that there was from one quartile to another was calculated, followed by the variation between T initial and T final, in order to calculate the variation that had occurred following the treatment with diode lasers or the placebo session. A mean was also carried out for the symptomatological scores that the patients reported in each session.

A statistical analysis was performed, using P.A.S.T. software (version 3.14 updated in November 2016, Øyvind Hammer, D.A.T. Harper and P.D. Ryan). The statistical significance of the differences was checked with the Mann–Whitney U-test. The level of significance was set to *p* < 0.05. Differences with a *p*-value less than 0.05 were considered statistically significant.

## 3. Results

All the patients had a generalized burning mouth sensation. In the evaluation of the symptomatological score of the oral mucosa, a clear improvement was seen on the VRS and NRS of the linear type in all the patients enrolled (Table 1). Their average scores were 7 at the beginning of the treatment (T#1) and 3/5 at the end of the treatment (T#8), showing a decreasing trend over the sessions.

In the evaluation of the capillary bed by videocapillaroscopy, there were variations in the laser group patients that, in consideration of the evaluation criteria adopted at the baseline, are summarized in the tables (Table 2 and Table 3). The capillary oral bed of patients in the placebo group did not show any statistically significant difference (*p* > 0.05). The results obtained show that, after treatment with diode lasers, in the laser group, there was a reduction in the diameter of the capillary loop (and therefore in the vascular diameter) in all the laser patients treated and in all the irradiated regions (*p* < 0.05). As can be seen, in the buccal mucosa, the diameter of the capillary loop from T1 (before treatment) and T8 (after eight treatments) had a reduction of 3 μm; in the upper lip mucosa, the diameter of the capillary loop from T1 to T8 had a reduction of 3 μm; in the lower lip mucosa, there was a reduction from T1 to T8 of 3 μm; in the dorsal lingual surface, there was a reduction from T1 to T8 of 2 μm. An increase in capillary length was also obtained in all irradiated regions in the laser group patients (*p* < 0.05). As can be seen, in the buccal mucosa, the capillary length from T1 (before treatment) and T8 (after eight treatments) increased by 70 μm; in the upper lip mucosa, the capillary length from T1 to T8 increased by 60 μm; in the lower lip mucosa, there was an increase from T1 to T8 of 50 μm; in the dorsal lingual surface, there was an increase from T1 to T8 of 130 μm. Neither the density of the capillaries, nor their tortuosity, nor the capillary morphology, showed significant variations from the start of the treatment to the end (*p* > 0.05).

## 4. Discussion

BMS is a highly debilitating condition for those patients who are affected. The burning sensation sometimes affects the quality of life and also the normal stomatognathic functions [4,5].

BMS has traditionally been treated using drugs that intersected well with possible hypotheses directly or indirectly involving the altered “neurosensory sphere” of these patients; among these drugs we must mention, for example, benzodiazepines (clonazepam); tricyclic antidepressants (amitriptyline); antiepileptics (carbamazepine); serotonergic antidepressants (trazodone); and substances such as capsaicin, which are able to desensitize nociceptive fibers [20,21,22,23,24,25,26].

In recent years, diode laser treatment, and therefore the administration of PBM, has been one of the most used therapeutic approaches for improving the quality of life of BMS patients; among other things, the method is very effective and almost completely risk free [10,13,14,15,16,19].

Some studies in the literature have shown a variation in the vascular pattern of patients with BMS [10,19]. From the analysis of the literature, we know that the most significant videocapillaroscopic data regards the variation of the diameter of the capillary loop, which perfectly coincides with the vasodilatation present in the phlogistic processes [10,19]. From the results of our study, it can be seen that, following treatment with diode lasers in the laser group patients, some data vary more frequently than others and in well-defined time periods, i.e., parameters such as the length of the capillary loop and the diameter of the capillary loop. We observed that at the moment of maximum reduction of the burning sensation (which the patients referred to us using the VAS and NRS scales) there was an increase in capillary length and a decrease in capillary diameter. To further substantiate this, it was seen that at those times when patients reported high VAS and NRS values, there was also an increase in capillary diameter and a decrease in capillary length.

Patients enrolled in the two groups showed a significant reduction in symptoms. After a period of 60 days, however, some patients showed a recurrence of the burning sensation, while others continued to report an absence or minimal presence of the burning sensation. The patients showing a relapse were those belonging to the placebo group; the patients showing a remission were those belonging to the laser group. Similar to the conclusions of some studies in the literature, our research shows that laser therapy in patients could have a placebo effect, but this is only transient. Conversely, the patients in the laser group also had a stable modification of the vascular bed, at least during the observation period, and this was associated with a longer-lasting period of remission of the symptoms. This evidence is extremely important because it shows that PBM has real effects on the oral vascular bed of patients, and therefore PBM is not a placebo therapy. It also demonstrates that by managing to modify the vascular bed, this therapy is able to bring about symptomatic remissions in patients which are more stable and that last over time.

Considering also that BMS is a syndromic pathology, the remission or, in any case, the acceptability of the level of the burning sensation, certainly improves the relational life of these patients. In addition, there is no use of systemic pharmacological therapies which, although they can have an effect on the symptoms of the disease, can also have an undesirable effect on the general state of health of these patients.

All these biomolecular mechanisms ultimately result in a reduction of vasodilatation (objectifiable by videocapillaroscopic investigation with the reduction of capillary diameter) that, from a practical point of view, could mean the reduction of phlogosis of the oral mucosa of BMS patients.

Therefore, remission of the burning symptomatology could be correlated with the reduction of the capillary diameter (objectifiable through videocapillaroscopic investigation), which perfectly corresponds to the reduction of vasodilation, a situation typically found in the pathologies that have phlogosis at their base. Therefore, the effectiveness of the diode laser is proven by experimental protocols and for this reason should be part of normal protocols since it is less invasive than pharmacological therapies.

As reported in the literature, some studies have demonstrated that PBM influenced microcirculation, it stimulated the secretion of angiogenic proteins, and then it influenced the microvascular pattern. PBM has a positive effect with regards to collagen expression and the number of newly formed vessels [12].

The improvement in the symptoms resulting from the reduction of the capillary diameter (and therefore of the vasodilatation typical of inflammation) could be related to PBM. PBM could reduce phlogosis, and then the burning sensation. In addition, our study shows that the effects of PBM on BMS are not linked to a placebo effect, but to an objectifiable effect that can be seen through the observation of the oral vascular pattern of these patients, thus, helping to remove the aura of mystery that often surrounds subjects with BMS.

## Figures and Tables

**Table 1:** Visual analog scale (VAS) and numeric rating scale (NRS) parameters in the photobiomodulation therapy (PBM) and placebo groups.

	T #1		T #8		After Window Period	
	NRS	VAS	NRS	VAS	NRS	VAS
PBM group	7	very severe pain	3	low pain	3	low/moderate pain
PLACEBO group	7	very severe pain	5	moderate pain	7	severe pain

**Table 2:** Summarized results on buccal mucosa, upper lip, lower lip, and dorsal lingual surface in the photobiomodulation group.

Variables	Before Treatment Buccal Mucosa	After Treatment Buccal Mucosa	Before Treatment Upper Lip	After Treatment Upper lip	Before Treatment Lower lip	After Treatment Lower lip	Before Treatment Dorsal Lingual Surface	After Treatment Dorsal Lingual Surface
*Length, μm*	311.25	383	297.2	359.3	281.5	334.25	286.1	417.5
*Diameter, μm*	12.625	9.5	10.78	7.25	12.64	9.5	11.6	9.25
*Density, n/mm^3^*	19.5	18.98	18.63	19.74	17.5	18.25	17.97	18.01
**Variables**								
*Tortuosity*	1	1	2	2	None	2	0	0
*Morphologiy*	None	Tortuous	Tortuous	Tortuous	Tortuous	Tortuous	Normal	Normal

**Table 3:** Summarized results on buccal mucosa, upper lip, lower lip, dorsal lingual surface in the placebo group.

Variables	Before Treatment Buccal Mucosa	After Treatment Buccal Mucosa	Before Treatment Upper Lip	After Treatment Upper Lip	Before Treatment Lower Lip	After Treatment Lower Lip	Before Treatment Dorsal Lingual Surface	After Treatment Dorsal Lingual Surface
*Length, μm*	307.20	305.40	285.2	290.7	280.4	283.77	296.1	300.5
*Diameter, μm*	13.48	12.96	10.44	11	11.33	11.5	12.5	11.9
*Density, n/mm³*	20	20.50	17.56	16.98	19.22	18.25	16.37	17.09
**Variables**								
*Tortuosity*	1	1	2	2	None	2	0	0
